# Development and validation of printed educational material for isotretinoin users^☆^^☆☆^

**DOI:** 10.1016/j.abd.2020.08.018

**Published:** 2021-05-29

**Authors:** Letícia Santos Dexheimer, Camila Boff, Cecília Cassal, Renan Rangel Bonamigo

**Affiliations:** a Service of Dermatology, Ambulatório de Dermatologia Sanitária, Rio Grande do Sul, RS, Brazil; b Postgraduate Program in Pathology, Brazil; c Hospital de Clínicas de Porto Alegre, Federal University of Rio Grande do Sul, Porto Alegre, RS, Brazil

Dear Editor,

Acne is a dermatosis that affects approximately 80% of adolescents.^1^ The treatment of the most severe forms involves the use of isotretinoin, a medication whose prescription requires strict control due to its high number of adverse effects. The use of printed educational materials during treatment with isotretinoin can increase user’s adherence and satisfaction, reducing side effects and complications.^2^

The present study developed an orientation manual for patients with acne who use isotretinoin and methodologically validated this material. The manual creation protocol consisted of two phases: the first phase involved the creation of educational material, based on the search for scientific articles in the databases between 1980 and 2019, using the descriptors: acne, isotretinoin, adherence, adverse effects, health education manuals. When creating the content, short phrases were used with simple, everyday language and in the active voice. The resulting manual was called “Patient Orientation Guide for Isotretinoin Use”; it has 16 pages, and its content was organized into 11 domains as follows:

1 What is isotretinoin?
2 What is Severe Inflammatory Acne?
3 What should I tell my physician before using isotretinoin?
4 How should I take isotretinoin?
5 What should I avoid while using isotretinoin?
6 What are the most common side effects of isotretinoin?
7 What should I immediately tell my physician while using isotretinoin?
8 Teratogenicity
9 Laboratory tests
10 Safe drug storage
11 Frequently asked questions

In the second phase, the educational manual was evaluated in two stages: validation by judges (Brazilian dermatologists who are acne experts), and validation by the target audience (patients).

There are controversies in the literature regarding the number and qualification of the judges. Lynn recommends a minimum of five and a maximum of ten people participating in this process.^3^ Pasquali points out that the number of six to twenty specialists is recommended for the validation process.^4^ In the first stage, nine judges were included, following the classification system described by Joventino (2010), which requires a minimum score of five, considering the academic degree and qualification in the area of ​​interest (Table 1). The specialists received the free and informed consent form and a questionnaire to be completed online, adapted from Galdino’s instrument (2019), containing closed questions about information included in the manual regarding the following: objectives, relevance, structure and presentation.^5^ In the second stage, after considering the judges’ suggestions, a new version of the manual was created, aimed at the target audience. Thirty patients were selected from Ambulatorio de Dermatologia Sanitaria, Rio Grande do Sul and Hospital de Clinicas de Porto Alegre.

**Table 1:** Selection criteria for judges of content (experts/researchers).

Judges of content	Score
Thesis or dissertation in the area of interest	2 points/thesis
Author of at least one study published in a journal indexed in the area of interest	1 point/study
Participation in research groups/projects that included the topic of the area of interest	1 point
Having been a member of a board of examiners of theses, dissertations or undergraduate monographs or specialization involving the area of interest	1 point
Teaching experience in a discipline in the area of interest	1 point/year
Practical experience with acne patients	0.5 point/year
Advisor of a thesis, dissertation or monograph in the area of interest	0.5 point/thesis

^a^Area of interest: Acne.

The criteria for selecting patients with acne were: age ≥14 years, isotretinoin use up to the third month of treatment, and adequate level of education for reading and understanding the material. The patients had to sign the free and informed consent form, and answer a questionnaire about the organization, style of writing, appearance and motivation of the educational material.

The study was approved by the Research Ethics Committee of institutions Ambulatorio de Dermatologia Sanitaria and Hospital de Clinicas de Porto Alegre with registers 3,389,244 and 3,584,111, respectively.

The Content Validity Index (CVI) was used to perform the interpretation and analysis of the data, which measures the proportion of judges who are in agreement about the instrument and its items.^6^ This method uses a Likert-type scale, with scores ranging from one to four. To assess relevance/representativeness, the answers may include^3^:

1 = not relevant, or not representative;

2 = needs further revision to be representative;

3 = needs a little revision to be representative;

4 = relevant or representative.

The index is calculated by adding the sum of the agreement on the items which got scores “3” and “4” by the specialists, divided by the total number of responses.^7^ Items that received a score of “1” or “2” must be reviewed or eliminated. In the case of six or more judges, an acceptable agreement rate of no less than 0.78 is recommended.^7^ In the analysis of the data evaluated by the target audience, items with a minimum level of agreement of 75% were considered validated.

Table 2 shows the evaluation of the manual by the judges regarding the objectives, structure, presentation and relevance of the manual. All items were validated, which conferred a Content Validity Index (CVI) of 0.88 for the proposed objectives, a CVI of 0.88 for structure and presentation and a CVI of 1.0 for relevance. There were some suggestions for changes in color, pictures and text.

**Table 2:** Evaluation of the judges of content regarding objectives, structure/presentation and relevance of the manual.

Questionnaire	Inadequate	Partially adequate	Adequate	Totally adequate	CVI
**1. Evaluation of the judges of content regarding the objectives of the manual.**					
1.1. The instrument is consistent with the needs of patients using isotretinoin	0	0	2	7	1.0
1.2. It should circulate in the scientific field in the dermatology area	0	1	2	6	0.88
1.3. The objective is evident, facilitating quick understanding of the material	0	0	1	8	1.0
**2. Evaluation of the judges of content regarding the structure and presentation of the manual.**					
2.1 The educational material is suitable to guide isotretinoin users	0	0	3	6	1.0
2.2 The messages are presented clearly and objectively	0	1	1	7	0.88
2.3 The presented information is scientifically correct	0	1	1	7	0.88
2.4 There is a logical sequence in the proposed content	0	0	0	9	1.0
2.5 The material is adequate for the sociocultural level of the proposed target audience	0	0	2	7	1.0
2.6 The information is well structured in agreement and spelling	0	1	0	8	0.88
2.7 The writing style corresponds to the level of knowledge of the target audience	0	0	3	6	1.0
2.8 The cover and back cover information is consistent	0	0	2	7	1.0
2.9 The illustrations are expressive and sufficient	0	1	2	6	0.88
2.10 The number of pages is adequate	0	0	3	6	0.88
2.11 The size of the title and topics is adequate	0	1	1	7	0.88
2.12 The level of difficulty of the content is adequate for the patients’ understanding	0	0	3	6	1.0
2.13. The text and/or pictures interact with the reader	0	0	2	7	1.0
**3. Evaluation of the judges of content regarding the relevance of the manual.**					
3.1 The topics depict key aspects that should be reinforced for isotretinoin users	0	0	2	7	1.0
3.2 The material proposes to the patients the acquisition of knowledge about isotretinoin use	0	0	2	7	1.0
3.3 The material addresses the necessary subjects about the risks of using the medication	0	0	2	7	1.0
3.4 It is adequate to be used by any health professional in their educational activities	0	0	2	7	1.0

Table 3 shows the evaluation of the manual by the patients. Of the thirty patients, 63% were aged between 15 and 20 years; 30% between 21 and 25 years; and 6.7% between 26 and 31 years. Most patients were males (56.6%), single (93.3%), had more than ten years of schooling (56.6%), had had acne for a period of less than or equal to five years (70%), and had undergone previous treatment with oral or topical medication (56.6%).

**Table 3:** Evaluation of the booklet by patients regarding the organization, writing style, presentation and motivation.

	Positive answers		Negative or indifferent answers		CVI
	n	%	n	%	
**1. Organization**					
1.1 Did the cover draw your attention?	25	83.3	5	16.6	0.83
1.2 Is the content sequence adequate?	30	100	0	0	1.0
1.3 Is the structure of the educational booklet organized?	30	100	0	0	1.0
**2. Writing style**					
2.1 As for the understanding of the phrases, they are: easy/difficult to understand	30	100	0	0	1.0
2.2 The written content is: clear/confusing	30	100	0	0	1.0
2.3 The text is interesting/uninteresting	30	100	0	0	1.0
**3. Presentation**					
3.1 The illustrations are: simple/complicated	29	96.6	1	3.3	0.967
3.2 Are the illustrations used to complement the text? Yes/No	29	96.6	1	3.3	0.967
3.3 Do the pages look organized? Yes/No	28	93.3	1	6.6	0.933
**4. Motivation**					
4.1 In your opinion, will any patient using isotretinoin who reads this booklet understand what it is about?	29	96.6	1	3.3	0.967
4.2 Did you feel motivated to read the booklet to the end?	29	96.6	1	3.3	0.967
4.3 Does the booklet address the issues necessary to solve the doubts of people who use isotretinoin?	29	96.6	1	3.3	0.967
4.4 Did the educational booklet suggest or guide you to act or change your behavior while using isotretinoin?	21	70	9	30	0.700

The material was positively assessed and validated by the patients regarding all aspects: organization, style and quality of the writing and three items of motivation, with the CVI always equal or very close to 1.0. One item related to motivation had a lower CVI (0.70) and was not validated by the patients (the one that addressed whether the manual would influence behavior change during isotretinoin use). It is possible that the quality of the information provided to patients by the two Dermatology Services where the study was carried out was the cause of the non-validation of this item, since the patients had probably already received guidance on the behavior while using isotretinoin.

The 16-page manual, validated by judges and patients, is illustrated in Fig. 1, in reduced size. The project developed easy-to-understand material, written in simple language, addressing the issues necessary to guide patients in relation to isotretinoin use, based on a sample of SUS-Brasil users.

**Figure 1:**
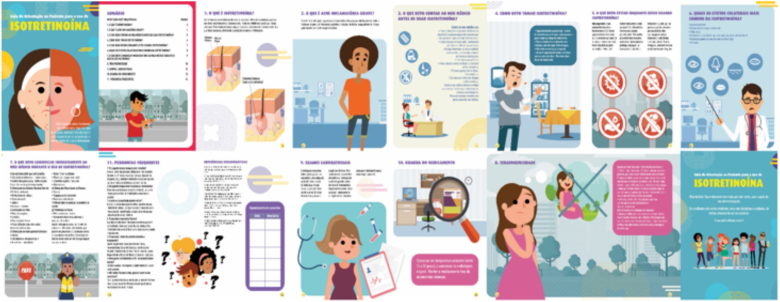
Complete manual, after validation (reduced size). 1. Cover; 2. Summary; 3. What is isotretinoin? ; 4. What is severe inflammatory acne? 5. What should I tell my physician before using isotretinoin? 6. How should I take isotretinoin? ; 7. What should I avoid while using isotretinoin? ; 8. What are the most common side effects of isotretinoin? ; 9. What should I immediately tell my physician while using isotretinoin? ; 10. Teratogenicity; 11. Laboratory tests; 12. Safe drug storage; 13. Frequently asked questions; 14. Back cover.

## Financial support

None declared.

## Authors’ contributions

Letícia Santos Dexheimer: Statistical analysis; design and planning of the study; drafting and editing of the manuscript; collection, analysis, and interpretation of data; intellectual participation in the propaedeutic and therapeutic conduct of the studied cases; critical review of the literature; critical review of the manuscript.

Camila Boff: Design and planning of the study; drafting and editing of the manuscript.

Cecília Cassal: Approval of the final version of the manuscript; design and planning of the study; drafting and editing of the manuscript; effective participation in research orientation.

Renan Rangel Bonamigo: Approval of the final version of the manuscript; design and planning of the study; drafting and editing of the manuscript; effective participation in research orientation; critical review of the manuscript.

## Conflicts of interest

None declared.
